# Multiple mechanisms contributing to ciprofloxacin resistance among Gram negative bacteria causing infections to cancer patients

**DOI:** 10.1038/s41598-018-30756-4

**Published:** 2018-08-16

**Authors:** Samira M. Hamed, Walid F. Elkhatib, Hadir A. El-Mahallawy, Mai M. Helmy, Mohamed S. Ashour, Khaled M. A. Aboshanab

**Affiliations:** 1 Department of Microbiology and Immunology, Faculty of Pharmacy, October University for Modern Sciences and Arts, 6th of October, Giza, Egypt; 2 Department of Microbiology and Immunology, Faculty of Pharmacy, Ain Shams University, African Union Organization St. Abbassia, Cairo, 11566 Egypt; 3 Department of Clinical Pathology, National Cancer Institute, Cairo University, Cairo, Egypt; 4 Department of Microbiology and Immunology, Faculty of Medicine, Zagazig University, Zagazig, Egypt; 5 Department of Microbiology and Immunology, Faculty of Pharmacy, Al-Azhar University, Cairo, Egypt

## Abstract

Fluoroquinolones have been used for prophylaxis against infections in cancer patients but their impact on the resistance mechanisms still require further investigation. To elucidate mechanisms underlying ciprofloxacin (CIP) resistance in Gram-negative pathogens causing infections to cancer patients, 169 isolates were investigated. Broth microdilution assays showed high-level CIP resistance in 89.3% of the isolates. Target site mutations were analyzed using PCR and DNA sequencing in 15 selected isolates. Of them, all had *gyrA* mutations (codons 83 and 87) with *parC* mutations (codons 80 and 84) in 93.3%. All isolates were screened for plasmid-mediated quinolone resistance (PMQR) genes and 56.8% of them were positive in this respect. Among PMQR genes, *aac(6′)-Ib-cr* predominated (42.6%) while *qnr* genes were harbored by 32.5%. This comprised *qnrS* in 26.6% and *qnrB* in 6.5%. Clonality of the *qnr*-positive isolates using ERIC-PCR revealed that most of them were not clonal. CIP MIC reduction by CCCP, an efflux pump inhibitor, was studied and the results revealed that contribution of efflux activity was observed in 18.3% of the isolates. Furthermore, most fluoroquinolone resistance mechanisms were detected among Gram-negative isolates recovered from cancer patients. Target site mutations had the highest impact on CIP resistance as compared to PMQRs and efflux activity.

## Introduction

Fluoroquinolones (FQs) are currently the primary antibiotics for antimicrobial prophylaxis in immunosuppressed cancer patients with the attendant risk of infection caused by the cytotoxic chemotherapy or the disease itself^1,2^. The main argument against this practice has been the selection of resistance to FQs. Cross-resistance to other antimicrobial agents may also develop with the emergence of multidrug resistant strains compromising the effectiveness of both prophylactic and empiric antibiotic regimens^3,4^.

FQs resistance is primarily caused by mutational alterations in target enzymes through stepwise mutations in the quinolone resistance-determining regions (QRDRs) of the DNA gyrase genes (*gyrA* and *gyrB*) and/or topoisomerase IV genes (*parC* and *parE*). Other mutations may occur in regulatory genes that control the expression of the outer membrane proteins (OMPs) and efflux pumps^5^. Plasmid-mediated quinolone resistance (PMQRs) is mediated by the quinolone-resistance protein (QNR) later named QnrA1. It is a pentapeptide repeat family protein that protects the target enzymes from quinolones action. Till date, seven Qnr proteins have been identified; QnrA, QnrB, QnrS, QnrC, QnrD, QnrVC and QnrE, with numerous genetic variants^6^. The second PMQR mechanism involves a mutant aminoglycoside-modifying enzyme (AAC(6′)-Ib-cr) that is capable of modifying certain quinolones, including ciprofloxacin (CIP) and norfloxacin, by adding an acetyl group thereby reducing their antibacterial activity^7^. The third mechanism of PMQR is the active efflux pumps QepA and oqxAB. QepA is a proton-dependent transporter belonging to the major facilitator superfamily that causes hydrophilic quinolone resistance^8^, while OqxAB is a novel transmissible resistance-nodulation-division (RND) multidrug efflux pump that was found to reduce susceptibility to CIP and nalidixic acid^9^.

Some studies worldwide have previously investigated the effect of CIP prophylaxis on the emergence of resistant bacteria in cancer patients^10–12^, but its impact on various mechanisms of resistance was not deliberately addressed. Accordingly, the aim of the current study was to elucidate the contribution of different mechanisms of CIP resistance among CIP non-susceptible Gram negative bacteria causing infections to cancer patients attending a tertiary cancer hospital in Cairo, Egypt during the study period.

## Results

### Susceptibility to ciprofloxacin

CIP MIC values of the tested clinical isolates ranged from 2–256 mg/L with MIC_50_ of 64 mg/L. High-level CIP resistance (MIC ≥ 32 mg/L) was evident in the majority of isolates (89.3%), most commonly among non-fermenter isolates (26/28, 92.9%), *E. coli* (74/80, 92.5%), *K. pneumoniae* (46/54, 85.2%), then other species (5/7, 71.4%). CIP MIC distribution and MIC_50_ among the isolates of various species are shown in Table 1.

**Table 1:** CIP MIC_50_ and MIC distribution among isolates of various species recovered from cancer patients.

Bacterial species	CIP MIC (mg/L)								CIP MIC_50_ (mg/L)
	2 (%)	4 (%)	8 (%)	16 (%)	32 (%)	64 (%)	128 (%)	256 (%)	
*E. coli* (n = 80)	1.3	0.0	1.3	5.0	8.8	32.5	33.8	17.5	128
*K. pneumoniae* (n = 54)	5.6	1.9	3.7	3.7	24.1	18.5	31.5	11.1	64
Non fermenters (n = 28)	0.0	3.6	0.0	3.6	21.4	25.0	39.3	7.1	64
Other species (n = 7)	14.3	14.3	0.0	0.0	28.6	14.3	14.3	14.3	32
Total isolates (n = 169)	3.0	1.8	1.8	4.1	16.6	26.0	33.1	13.6	64

Other species, *C. freundii, E. aerogenes, E. cloaceae, K. oxytoca* and *P. mirabilis*; Non-fermenters, *A. baumannii* and *P. aeruginosa*.

CIP MIC_50_, ciprofloxacin MIC at which 50% of the isolates were inhibited.

### Target-affecting resistance mechanisms

DNA sequencing of the QRDRs of *gyrA* and *parC* genes from 15 isolates revealed at least one missense mutation, including a *gyrA* mutation, in all isolates. Codon 83 of the *gyrA* gene had the highest incidence of such mutations (15/15, 100%) with the resulting substitutions: Ser/Leu, Ile, Tyr or Phe in all species except *P. aeruginosa* where threonine was substituted by isoleucine. This was accompanied by another mutation at codon 87 in 40% (6/15) of the isolates (Asp/Asn, Tyr or Ala). Amino acid substitutions in ParC were evident in 93.3% (14/15) of the isolates at codon 80 (13/15, 86.7%) including: Ser to Ile or Leu substitutions and codon 84 where Lys was substituted for Glu in one isolate (6.7%). Detailed information on the isolates tested for target site mutations are shown in Table 2. Double mutations (1 *gyrA/*1 *parC*) were the most frequently encountered among the tested isolates (8/15, 53.3%) followed by three mutations (2 *gyrA*/1 *parC*) found in 40% (6/15), while only 6.7% (1/15) showed a single *gyrA* mutation.

**Table 2:** Distribution of CIP resistance mechanisms including target site mutations, PMQR determinants, and efflux activity among the tested clinical isolates.

Isolate No.	Species	Specimens	PMQR genes	CIP MIC (mg/L)	Efflux Activity	GyrA^83^	GyrA^87^	ParC^80^	ParC^84^
26	*E. coli*	Stools	−	256	−	S-L	D-Y	S-I	WT
82a	*E. coli*	Blood	*aac(6′)-Ib-cr*	128	−	S-L	D-N	S-I	WT
5	*E. coli*	Blood	−	128	−	S-L	D-N	S-I	WT
3	*K. pneumoniae*	Blood	*qnrB/aac(6′)-Ib-cr*	128	−	S-Y	D-A	S-I	WT
116	*K. pneumoniae*	Blood	*qnrS*	256	−	S-Y	D-N	S-I	WT
117	*K. pneumoniae*	Blood	*−*	256	+	S-F	D-N	S-I	WT
141	*K. pneumoniae*	Blood	*qnrB/qnrS/aac(6′)-Ib-cr*	32	*−*	S-Y	WT	WT	WT
304	*K. pneumoniae*	Blood	*qnrS/aac(6′)-Ib-cr*	64	*−*	S-I	WT	S-I	WT
254b	*P. mirabilis*	Pus	*qnrA*	8	−	S-I	WT	S-I	WT
205b	*P. aeruginosa*	Sputum	*qnrB*	32	−	T-I	WT	S-L	WT
206c	*P. aeruginosa*	Sputum	*−*	32	−	T-I	WT	S-L	WT
256b	*P. aeruginosa*	Pus	*−*	32	−	T-I	WT	S-L	WT
305	*A. baumannii*	Blood	*qnrS*	64	−	S-L	WT	S-L	WT
251b	*A. baumannii*	W. swabs	*−*	128	+	S-L	WT	WT	E-K
284	*A. baumannii*	Blood	*−*	128	+	S-L	WT	S-L	WT

W. swab; wound swab, PMQR, Plasmid-mediated quinolones resistance; CIP MIC, ciprofloxacin MIC (mg/L); The superscripts of protein names indicate the codon number (*E. coli* numbering); WT, wild type protein.

The Kruskal-Wallis test showed that the distribution of CIP MIC across the groups of isolates carrying different numbers of target site mutations is significantly different (*P*-value = 0.013). The highest MIC_50_ (128 mg/L) was shown by the group of isolates carrying 3 target site mutations while those having 2 mutations had an MIC_50_ of 32 mg/L. One target site mutation was observed in a single isolate that showed CIP MIC of 32 mg/L.

### Prevalence of PMQR genes

PMQR determinants were detected in 56.8% of the clinical isolates. They were more statistically abundant among Enterobacteriaceae than non-fermenter isolates (66.7% versus 7.1%, *P*-value < 0.001). Among Enterobacteriaceae isolates, the genes *aac(6′)-Ib-cr, qnrS*, *qnrB* and *qnrA* were present in 51.1%, 31.2%, 7.1% and 0.7%, respectively. A total of 37.6% of the isolates were positive for *qnr* genes. The *aac(6′)-Ib-cr* variant was evident in 87.8% of the *aac(6′)-Ib*-positive Enterobacteriaceae isolates (72/82) while coexistence of both was observed in 7.3% of them. The *qnr* genes and *aac(6′)-Ib-cr* were detected in combination in 21.9% of the isolates. No isolates carrying the *qepA* gene were identified while *oqxAB* genes were only detected in *Klebsiella* spp isolates (96.4%). Two *K. pneumoniae* isolates co-harbored *qnrS*, *qnrB*, *aac(6′)-Ib-cr, oqxA* and *oqxB* genes.

Of all tested PMQR determinants, only *qnrS* and *qnrB* genes were found in non-fermenter isolates. One *A*. *baumannii* isolate (4.3%) harbored the *qnrS* gene and the *qnrB* gene was carried by one *P. aeruginosa* isolate (20.0%). The prevalence of different PMQR determinants with respect to bacterial species is shown in Table 3.

**Table 3:** Prevalence of PMQR genes in various bacterial species.

PMQR determinants	Number of isolates (%)				Total isolates (n = 169)
	*E. coli* (n = 80)	*K. pneumoniae* (n = 54)	Other species (n = 7)	Non-fermenters (n = 28)	
*qnrS*	5(6.2)	14(25.9)	0(0.0)	1(3.6)	20(11.8)
*qnrB*	0(0.0)	2(3.7)	0(0.0)	1(3.6)	3(1.8)
*qnrA*	0(0.0)	0(0.0)	1(14.3)	0(0.0)	1(0.6)
*aac(6′)-Ib-cr*	35(43.8)	5(9.3)	1(14.3)	0(0.0)	41(24.3)
*qnrS/aac(6′)-Ib-cr*	14(17.5)	9(16.7)	0(0.0)	0(0.0)	23(13.6)
*qnrB/aac(6′)-Ib-cr*	0(0.0)	6(11.1)	0(0.0)	0(0.0)	6(3.6)
*qnrS/qnrB/aac(6′)-Ib-cr*	0(0.0)	2(3.7)	0(0.0)	0(0.0)	2(1.2)
Total PMQR determinants	54(67.5)	38(70.4)	2(28.6)	2(7.1)	96(56.8)

PMQR, plasmid-mediated quinolones resistance; Other species, *C. freundii, E. aerogenes, E. cloaceae, K. oxytoca*, and *P. mirabilis*; non-fermenters, *A. baumannii* and *P. aeruginosa*. Percentages were calculated relative to the number of isolates in each species.

Although higher CIP MIC_50_ was observed among PMQR-positive isolates than PMQR-negative isolates (128 versus 64 mg/L, respectively), the distribution of CIP MIC across the two groups was not significantly different (Mann Whitney U test, *P*-value = 0.140).

### Clonal Relatedness of *qnr*-positive isolates

ERIC-PCR was performed on 33 *qnr*-positive *K. pneumoniae* isolates and showed 32 banding patterns classified into 22 ERIC types (K1-K22) based on a similarity percentage of 85% or more (see Supplementary Fig. S1). On the other hand, greater diversity was evident among 19 *qnr*-positive *E. coli* isolates that showed 19 amplification patterns comprising 14 ERIC types (E1-E14) (see Supplementary Fig. S1). Significant association between ERIC types and PMQR determinants was observed in *K. pneumoniae* isolates (Fishers Exact Test, *P* = 0.005). In contrast such association was not evident in *E. coli* isolates (Fishers Exact Test, *P* = 0.674).

### Efflux activity

**Figure 1:**
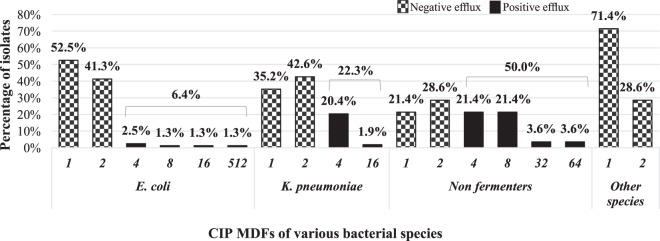
CIP MDF values and prevalence of isolates showing active efflux activity among various species. CIP MDF, ciprofloxacin MIC decrease factor; other species, other Enterobacteriaceae including: *C. freundii*, *E. aerogenes*, *E. cloaceae*, *K. oxytoca* and *P. mirabilis*; non-fermenters, *A. baumannii* and *P. aeruginosa*. Isolates of CIP MDF ≥ 4 are considered to have active efflux activity.

Using the efflux pump inhibitor, four fold or more CIP MIC reduction (MDF value of more ≥4) was observed in 18.3% of the isolates, most commonly in non-fermenters (50.0%). On the other hand, 66 isolates (39.1%) exhibited a MDF value of 2, while CIP MIC of 72 isolates (42.6%) did not show any difference by combining CCCP. The distribution of MDF values among various species as well as the prevalence of isolates showing active efflux activity are shown in Fig. 1.

Although efflux activity was associated with higher CIP MIC_50_ (128 versus 64 mg/L), the distribution of CIP MIC was found to be the same across the groups of isolates showing positive efflux activity and others (Mann Whitney U test, *P*-value = 0.406).

## Discussion

The current study was conducted with the aim of elucidating CIP resistance acquisition mechanisms among Gram negative pathogens causing infections to cancer patients, a population to whom FQs are widely prescribed for prophylaxis of bacterial infections. For this purpose, 169 Gram-negative isolates recovered from different clinical specimens from infected cancer patients in Egypt were investigated. Only CIP-resistant isolates were included in the current study. Broth microdilution assays revealed high-level CIP resistance (CIP MIC ≥ 32 mg/L) in the majority of isolates (89.3%). Such a noticeable prevalence of high-level resistance was also reported among Enterobacteriaceae isolates recovered from a tertiary hospital in Poland^13^.

The acquisition of FQs resistance is known to be most attributable to mutations in the target enzymes-coding genes. A genetic analysis based on the QRDR sequences of *gyrA* and *parC* genes of selected isolates of various Gram negative genera revealed that all strains carried point (missense) mutations in *gyrA* codon 83 with simultaneous mutations at codon 87 in 40.0% of the isolates. Such mutation sites are the most commonly encountered worldwide^5,14^. Their high mutability is likely due to being located near the active site of the DNA gyrase^15^. On the other hand, only 93.3% carried *parC* gene mutations (codons 80 or 84). This finding supports the previous hypothesis that topoisomerase IV mutations may not occur in Gram negative bacteria unless the sensitivity of the DNA gyrase to FQs has been reduced by missense mutations^5,14^.

All tested non-fermenter isolates showed two target site mutations (1 *gyrA*/1 *parC*). Such mutation pattern was reported by previous studies as the most common among CIP-resistant *A. baumannii*^16–18^ and *P. aeruginosa* isolates^19–21^. Nevertheless, single *gyrA* mutations were sufficient to cause clinically significant levels of resistance in both species according to some studies^19–24^. Triple mutations (2*gyrA*/1*parC*) were also previously reported^16^.

Consistent with the literature^5,14^, our results showed a substantial increase in CIP resistance levels with accumulating mutations in one or both target enzymes. Notably, a wide variability in CIP MIC was shown by the isolates carrying two target site mutations, likely due to the contribution of other resistance mechanisms. Of particular interest was the finding that double mutations (1*gyrA*/1*parC*) were sufficient to confer high-level CIP resistance among non-fermenter isolates. In contrast, three mutations were carried by 75.0% of Enterobacteriaceae isolates showing high-level CIP resistance. This is probably due to the intrinsic resistance of non-fermenters to antibacterial agents compared to members of Enterobacteriaceae as a result of low permeability and/or over expression of some efflux pumps^5,25^.

Despite their inability to confer FQs resistance, PMQRs play an important role in the acquisition of clinical resistance to FQs. The low-level quinolone-resistance phenotypes conferred by PMQR genes may allow some low-fitness mutants below the resistance breakpoint to evolve clinical resistance with just one or two mutations^26^. Most importantly, PMQRs can spread horizontally among different Gram negative species^5^. PCR screening of PMQR genes in the current study revealed their carriage by 66.7% of Enterobacteriaceae isolates. Such a high prevalence reported here and by other studies performed worldwide among FQ-resistant Enterobacteriaceae isolates, reflects their crucial role in acquisition of FQs resistance. In contrast, lower prevalence of PMQRs was reported in CIP-resistant Enterobacteriaceae isolates from Poland (22.8%)^13^ and FQ-resistant *E. coli* from Taiwan (14.9%)^27^. Consistent with other studies^13,28^, PMQR genes were more frequently detected among the isolates of *K. pneumoniae* (70.4%) than *E. coli* (67.5%) and other Enterobacteriaceae species (28.6%).

Although higher frequencies of PMQRs are expected from isolates preselected for extended-spectrum β-lactamases (ESBLs) production^29^, lower PMQRs prevalence was reported among ESBL-producer *K. pneumoniae* from Kuwait (30.6%)^30^, *E. coli* from Tunisia (50.0%)^28^ and Argentina (62.5%)^31^. Whether the high prevalence of PMQR-producers among our collection is linked to their recovery from cancer patients or due to their high regional prevalence, this was further analyzed by reviewing other Egyptian studies. In a recent study by El-badawy *et al*., *qnr* genes (*qnrB* and *qnrS*) were detected in 91.2% of FQ-resistant *K. pneumoniae* isolates. This was much higher than their prevalence among the same species in our collection (61.1%). On the other hand, the prevalence of *qnr* genes in *E. coli* isolates in the current study was comparable (23.8%) to that reported, in ESBL-producer *E. coli*, by another Egyptian study (26.6%), while *aac(6′)-Ib-cr* was more frequently detected in our collection (61.2% versus 23.3%)^32^.

Enzyme modification through AAC(6′)-Ib-cr was the most prevalent PMQR mechanism among Enterobacteriaceae isolates in the current study (51.5%). A similar predominance of this mechanism was reported by other studies^13,33,34^. Higher prevalence of the *aac(6′)-Ib-cr* variant compared to the wild type gene was evident in our collection (87.8%) as well as previous studies^34–36^. This is possibly due to its wider spectrum of activity that uniquely encompasses two different classes of antimicrobial agents including quinolones and aminoglycosides^37^. Of the *qnr* genes, *qnrB* seems to be the most prevalent worldwide^29^. In our study, however, *qnrS* was more frequently detected than *qnrB* (31.2% versus 7.1%). The *qnrA* gene, the least frequent of the *qnr* genes, was described in a single *P. mirabilis* isolate. Nevertheless, it was absent in recent worldwide surveillance studies^28,31,36,38^.

Concerning plasmid-encoded efflux pumps, QepA*-*producers were not identified. Similar findings were described in several studies in Tunisia^28^, Argentina^39^, and France^36^. In contrast, *qepA* gene was detected among *K. pneumoniae* isolates from non cancer patients in Egypt^40^ and *E. coli* isolates from Algeria^41^ and Pakistan^33^. On the other hand, *oqxAB* genes were only detected among members of *Klebsiella* spp. in which the *oqxAB* operon is mostly located on the chromosome and has no correlation with CIP MIC, likely due to different expression levels^42^. Accordingly, *oqxA* and *oqxB*-positive *Klebsiella* spp. isolates were not considered among the PMQR-positive isolates in the current study.

A relatively few number of studies worldwide were concerned with detection of PMQR genes among non-fermenter isolates in which they are known to be rare. In the current study, a significantly lower prevalence of PMQRs was found among non-fermenter than Enterobacteriaceae isolates (7.1% versus 66.7%, *P* < 0.001) suggesting higher contribution of other resistance mechanisms^5^. The *qnrS* and *qnrB* genes were detected in 4.3% of *A. baumannii* and 20.0% of *P. aeruginosa* isolates, respectively. A similar prevalence of *qnrB* gene was reported from Poland among *P. aeruginosa* isolates^43^, other PMQR genes were also previously reported^44–46^. In *A. baumannii*, *qnrS* and *qnrB* were reported in China^47^ and *qnrA* gene was reported in Algeria^48^.

Although their carriage did not have a significant effect on CIP MIC among the tested isolates (*P* = 0.140), detection of PMQR genes in almost all tested species in our collection confirms their interspecies transferability. More alarming is the previous knowledge of their existence in multi-resistance plasmids linked to other resistance determinants^14,49^. In light of this, it should be stressed that carriage of PMQR determinants are of great public health concern owing to their ability to confer multidrug resistance to other potential recipient strains. Failure of empirical antimicrobial treatment of Gram negative infections, following CIP prophylaxis, in cancer patients is then inevitably expected.

To investigate the dissemination of *qnr* genes among the isolates of the same species, it was of interest to examine their clonal relatedness. Molecular characterization using ERIC-PCR showed a considerable genomic diversity among the *qnr*-positive isolates. Twenty two ERIC types were identified among 33*K. pneumoniae* isolates where up to seven isolates had the same ERIC type. Moreover, ERIC types were significantly related to PMQR determinants (*P* = 0.005), suggesting a low-level clonal dissemination. On the other hand, among 19 *E. coli* isolates, 14 ERIC types were evident without significant relation to PMQRs (*P* = 0.674) indicating that most of them were not clonal. This provides additional evidence on the horizontal transfer of PMQR genes described elsewhere rather than their clonal dissemination^13,30,50^.

Active efflux and/or reduced influx of FQs can reduce their cytoplasmic concentrations conferring FQs resistance^5^. To elucidate the involvement of efflux pumps in CIP resistance in our collection, CIP MIC was determined in the presence of sub-inhibitory concentrations of CCCP, a protonophore that reduces ATP production and increases bacterial cell membrane permeability^51^. Based on a fourfold or greater reduction in CIP MIC (MDF ≥ 4) as a criterion for significance^52^, efflux activity was evident in 18.3% of the isolates. Consistent with the literature^5^, this was significantly more frequent among non-fermenter than Enterobacteriaceae isolates (50.0% versus 12.1%, *P* < 0.001). The distribution of CIP MIC was found to be the same among the isolates showing positive efflux activity and others (*P* = 0.406). A possible explanation is that the effect of over active efflux pumps is often limited to about four- to eightfold increase in inhibitory concentrations (except for rare cases in our collection)^5^. Such effect may be overlooked when other mechanisms, particularly target site mutations, are established. Similar findings were reported by other studies as well^24,53^. The results of our study, however, demonstrate a loss of CIP resistance in 19.3% of the isolates showing active efflux activity by combining CCCP. In this context, the use of efflux pump inhibitors appears to be a promising strategy to restore antibacterial potency.

## Conclusion

The current study provides information on the mechanisms underlying CIP resistance among Gram negative isolates causing infections to cancer patients in Egypt. Being affected by the same resistance mechanisms, the results of the current study could be extrapolated to other FQs that are widely used for prophylaxis of bacterial infections in this population. The information provided herein showed high-level CIP resistance in the majority of isolates. Resistance was a result of a complex interplay between most of the known FQs resistance mechanisms each demonstrated in a high prevalence. The current results also provide additional evidence that chromosomal mutations in sequences encoding GyrA and ParC, detected in all tested isolates, play an essential role in CIP resistance. It is noteworthy that the dissemination of PMQR genes, with a high prevalence, was demonstrated in clonally unrelated isolates. Reduced antibiotic accumulation arising from the over expression of efflux pump systems was also evident but with low impact on CIP MIC. Failure of empirical treatments of Gram negative infections in cancer patients following CIP prophylaxis is a possible scenario and it would be attributed to the coexistence of other genes conferring resistance to different antimicrobial agents.

## Methods

### Bacterial isolates and susceptibility testing

A total of 169 Gram negative bacterial isolates showing reduced susceptibility to CIP were recovered from clinical specimens collected from infected cancer patients admitted to the National Cancer Institute (NCI), Cairo University in the period from November 2014 to July 2015. Informed consent for study participation was obtained from all patients. Bacterial isolates were recovered from different specimen types including; blood (103; 60.9%), wounds (16; 9.5%), sputum (14; 8.3%), pus (13; 7.7%), stools (8; 4.7%), urine (5; 3%), drain (4; 2.4%), bronchoalveolar lavage (4; 2.4%), and chest tubes (2; 1.2%). The isolates comprised *Escherichia coli* (n = 80), *Klebsiella pneumoniae* (n = 54), *Acinetobacter baumannii* (n = 23), *Pseudomonas aeruginosa* (n = 5), *Klebsiella oxytoca* (n = 2), *Enterobacter cloaceae* (n = 2), *Enterobacter aerogenes* (n = 1), *Proteus mirabilis* (n = 1) and *Citrobacter freundii* (n = 1).

VITEK^®^2 system (bioMérieux, Marcy-l′Étoile, France) was used for species identification and antimicrobial susceptibility testing as a part of the routine laboratory work in the hospital. All CIP non-susceptible Gram negative isolates (MIC ≥ 2 mg/L) were selected for the study. The minimum inhibitory concentration (MIC) of CIP was determined using the broth microdilution method in accordance with the Clinical and Laboratory Standards Institute (CLSI) guidelines^54^. *E. coli* ATCC 25922 was used as a quality control strain.

Approval of the study protocol was received from the Ethical Review Boards of each of Ain Shams University, October University for Modern Sciences and Arts and the National Cancer Institute, Cairo University. All methods were performed in accordance with the required guidelines and regulations.

### Genotypic detection of target-affecting resistance mechanisms

Fifteen isolates of different species were selected for analysis of mutations in the QRDRs of the *gyrA* and *parC* genes. Those comprised five *K. pneumoniae* isolates, three *E. coli* isolates, three *P. aeruginosa* isolates, three *A. baumannii* isolates, and a single *P. mirabilis* isolate. Specific primers (Table 4) for amplification of gene fragments encompassing the QRDR and flanking nucleotide sequences in different species were designed based on the wild-type gene sequences of corresponding standard strains published in the GenBank nucleotide sequence database (www.ncbi.nlm.nih.gov). Genomic DNA was extracted using Genomic DNA Purification Kit (Thermo Fisher Scientific, Waltham, MA, USA) according to the manufacturer’s instructions. Amplification was performed in a final volume of 25 μL containing 1 μL of each primer at a concentration of 12.5 μM, 12.5 μl MyTaq^TM^ Red Mix, and approximately 100 ng of chromosomal DNA. PCR reactions were performed using TAdvanced thermal cycler (Biometra, Germany) with an initial denaturing cycle at 95 °C for 5 min followed by 35 cycles of 95 °C for 30 s, annealing temperature for 30 s, and 72 °C for 1 min, with a final extension step at 72 °C for 5 min.

**Table 4:** Primers used for amplification of *gyrA* and *parC* genes from various species.

Target gene	Primer	Sequence (5′-3′)	Organism	Gene fragment	Product size (bp)	Ta (°C)
*gyrA*	GyrA-F	AGCGACCTTGCGAGAGAAAT	*E. coli, K. pneumoniae*	4–457	454	58
	GyrA-R	CCGTGCCGTCATAGTTATCAAC				
	GyrAPr-F	CATTGCCAGAGAAATCACACCAG	*P. mirabilis*	9–663	655	60
	GyrAPr-R	CGCAGCAGTCGGAAAATCAG				
	GyrAAc-F	AAACCTGTTCACCGTCGTGT	*A. baumannii*	118–658	541	66
	GyrAAc-R	TACCGCCTGTAGGGAAGTCA				
	GyrAPs-F	CACCGCCGTGTGCTTTATG	*P. aeruginosa*.	134–976	843	64
	GyrAPs-R	GGGTCTGGGCATAGAGGTTG				
*parC*	ParC-F	CGTGCGTTGCCGTTTATTG	*E. coli*	85–747	663	60
	ParC-R	ATCTTCTTTCTTCCACACCGC				
	ParCK-F	GGACAGGGCATTACCGTTTA	*K. pneumoniae*	81–554	474	60
	ParCK-R	AGGTTGTGCGGAGGAATATC				
	ParCPr-F:	ATGATGGTGTAGAGCGCCAA	*P. mirabilis*	17–770	754	57
	ParCPr-R	ACCGCACAACCCTCTTCTTT				
	ParCAc-F	CAGAAAACCGCTCTGTAGCC	*A. baumannii*.	26–944	919	57
	ParCAc-R	ACTGCTTCCGCATCAATAC				
	ParCPs-F	TCGATCTGAGCCTGGAAGG	*P. aeruginosa*.	14–419	406	60
	ParCPs-R	AGCAGCACCTCGGAATAGC				

Ta, annealing temperature.

All PCR products were purified using DNA Clean & Concentrator^TM^-25 Kit (Zymo Research, Orange, CA, USA) and sequenced by the Sanger method using an ABI 373 A DNA sequencer (PE Applied Biosystems, Life technologies Inc., CA, USA). The predicted amino acid sequences were analysed for amino acid changes by comparison to the wild-type GyrA and ParC sequences of the corresponding standard strains deposited in the GenBank at the National Center for Biotechnology Information website (http://www.ncbi.nlm.nih.gov/blast) using the BLAST tool.

### Screening of plasmid-mediated resistance mechanisms

Using multiplex PCR, all the isolates were screened for the PMQR genes; *qnrA, qnrB, qnrS*, and *aac(6*′*)-Ib*, while monoplex PCRs were used for the detection of *qepA*, *oqxA* and *oqxB* genes using gene specific primers listed in Table 5, as we described previously^55^. Plasmid DNA, extracted by a GeneJET Plasmid Miniprep Kit (Thermo Fisher Scientific, Waltham, MA, USA), was used as template for PCR reactions. The identity of the amplified genes was confirmed by DNA sequencing of representative PCR products of each gene and comparisons of the obtained sequences with those in the GenBank using the BLAST program (http://blast.ncbi.nlm.nih.gov) were performed. Harboring *aac(6*′*)-Ib-cr* gene was confirmed by the enzymatic digestion of all amplification products of the *aac(6*′*)-Ib* gene with *BseG*I (Thermo Fisher Scientific, Waltham, MA, USA). The presence of 269-bp and 211-bp DNA fragments indicated *aac(6*′*)-Ib*, whereas an undigested fragment was indicative of the *aac(6*′*)-Ib-cr* variant.

**Table 5:** Primers used for screening of PMQR genes.

Primer	Sequence (5′-3′)	Target Gene	Product size (bp)	Ta (°C)
QnrA-F	GCCCGCTTCTACAATCAAGT	*qnrA*	347	60
QnrA-R	GGCAGCACTATTACTCCCAAG			
QnrB-F	TATGGCTCTGGCACTCGTT	*qnrB*	193	60
QnrB-R	GCATCTTTCAGCATCGCAC			
QnrS-F	TCGGCACCACAACTTTTCAC	*qnrS*	255	60
QnrS-R	TCACACGCACGGAACTCTAT			
Aac(6′)-Ib-F	CTTGCGATGCTCTATGAGTGG	*aac(6*′*)-Ib*	480	60
Aac(6′)-Ib-R	GAATGCCTGGCGTGTTTGAA			
QepA-F	TCTACGGGCTCAAGCAGTTG	*qepA*	312	55
QepA-R	ACAGCGAACCGATGACGAAG			
OqxA-F	CTCTCCTTTCTGCTCGTCGG	*oqxA*	489	67
OqxA-R	AATAGGGGCGGTCACTTTGG			
OqxB-F	TAGTGCTGGTGGTGCTGGTA	*oqxB*	480	68
OqxB-R	GGGTAGGGAGGTCTTTCTTCG			

Ta, annealing temperature.

### Clonality analysis of *qnr*-positive isolates

All *qnr*-positive isolates of the same species were analyzed by ERIC-PCR using ERIC2 primer; 5′- AAGTAAGTGACTGGGGTGAGCG -3′ as described previously^56^. Amplification patterns were analyzed using GelCompar II software (Applied Math, Kortrijk, Belgium). Similarity clustering analyses were performed using UPGMA (unweighted pair group method with arithmetic mean) and Dice correlation coefficient with a position tolerance of 1.5%.

### Assessment of efflux pump activity

To investigate the role of efflux pumps in the development of CIP resistance, the MIC of CIP was determined in the presence of sub-inhibitory concentrations of the efflux pump inhibitor; carbonyl-cyanide-m-chlorophenylhydrazone (CCCP) (20 and 10 mg/L for Enterobacteriaceae and non-fermenters, including *A. baumannii* and *P. aeruginosa*, respectively). The MIC of CCCP in different Gram negative species was determined in twofold serial dilutions as described by the clinical and laboratory standards institute (CLSI) methodology for antimicrobial susceptibility testing^57^. A positive control was included to test the viability of different isolates in presence of CCCP alone. Subsequently, the MIC decrease factor (MDF) of each isolate was calculated according to the following formula; MDF = MIC_without CCCP_/MIC_with CCCP_ as described before^58^. An MDF value of 4 or more was considered as a significant effect due to efflux pump inhibition via CCCP.

### Nucleotide sequence accession numbers

The nucleotide sequences of *gyrA* and *parC* genes of some clinical isolates analyzed in the current study were submitted to GenBank nucleotide sequence database. Accession numbers are shown in Table 6.

**Table 6:** Accession numbers submitted to the NCBI of *gyrA* and *parC* fragments amplified from different isolates of various genera.

Species	Isolate No.	Gene	Accession
*Escherichia coli*	26	*gyrA*	MF991461
	82b	*gyrA*	MF991462
*Klebsiella pneumoniae*	117	*gyrA*	MG014723
	3	*gyrA*	MG471385
	116	*gyrA*	MG198059
	304	*gyrA*	MG198060
	141	*gyrA*	MG242342
*Proteus mirabilis*	254b	*gyrA*	MG198061
*Pseudomonas aeruginosa*	205b	*gyrA*	MG198062
*Acinetobacter baumannii*	251b	*gyrA*	MG198063
*Escherichia coli*	26	*parC*	MG242340
*Klebsiella pneumoniae*	116	*parC*	MG242341
*Pseudomonas aeruginosa*	205b	*parC*	MG242343
*Acinetobacter baumannii*	251b	*parC*	MG753556
	284	*parC*	MG753557

### Statistical analyses

All tests of significance were two-tailed, and statistical significance was defined at *P* ≤ 0.05. Statistical analyses were performed using IBM SPSS Statistics for Windows version 20.0 (IBM Corp., Armonk, NY, USA).

Categorical variables were analyzed using the Chi-square test or when expected cell counts were <5, Fisher’s exact test was used. The Gaussian distribution of continuous variables values was evaluated using the Shapiro-Wilk test. Comparisons of continuous variables were performed using Mann-Whitney U test, Kruskal-Wallis test, and Wilcoxon Signed Rank test as appropriate.

## Electronic supplementary material

Supplementary Figure S1
