# Supplementary material for: Multiple mechanisms contributing to ciprofloxacin resistance among Gram negative bacteria causing infections to cancer patients
Source: Sci Rep. 2018 Aug 16;8:12268. doi: 10.1038/s41598-018-30756-4 (PMC6095922; doi:10.1038/s41598-018-30756-4)
Supplement: Supplementary file 1 — Supplementary Figure S1 [file 41598_2018_30756_MOESM1_ESM.pdf]

# Multiple mechanisms contributing to ciprofloxacin resistance among Gram negative bacteria causing infections to cancer patients

**Samira M. Hamed[1], Walid F. Elkhatib[2*], Hadir A. El-Mahallawy[3], Mai M. Helmy[4], Mohamed S. Ashour[5] and Khaled M. A. Aboshanab[2]**

[1]Department of Microbiology and Immunology, Faculty of Pharmacy, October University for Modern Sciences and Arts, 6th of October, Egypt

[2]Department of Microbiology and Immunology, Faculty of Pharmacy, Ain Shams University, African Union Organization St. Abbassia, Cairo 11566, Egypt

[3]Department of Clinical Pathology, National Cancer Institute, Cairo University, Cairo, Egypt

[4]Department of Microbiology and Immunology, Faculty of Medicine, Zagazig University, Zagazig, Egypt

[5]Department of Microbiology and Immunology, Faculty of Pharmacy, Al-Azhar University, Cairo, Egypt

[*]Corresponding author: Prof. Dr. Walid F. Elkhatib

Postal address: [1]Department of Microbiology & Immunology, Faculty of Pharmacy, Ain Shams University, African Union Organization St. Abbassia, Cairo 11566, Egypt

Tel: +202-24051120, Fax: +202-24051107

Email: walid-elkhatib@pharma.asu.edu.eg

## Supplementary Figure S1

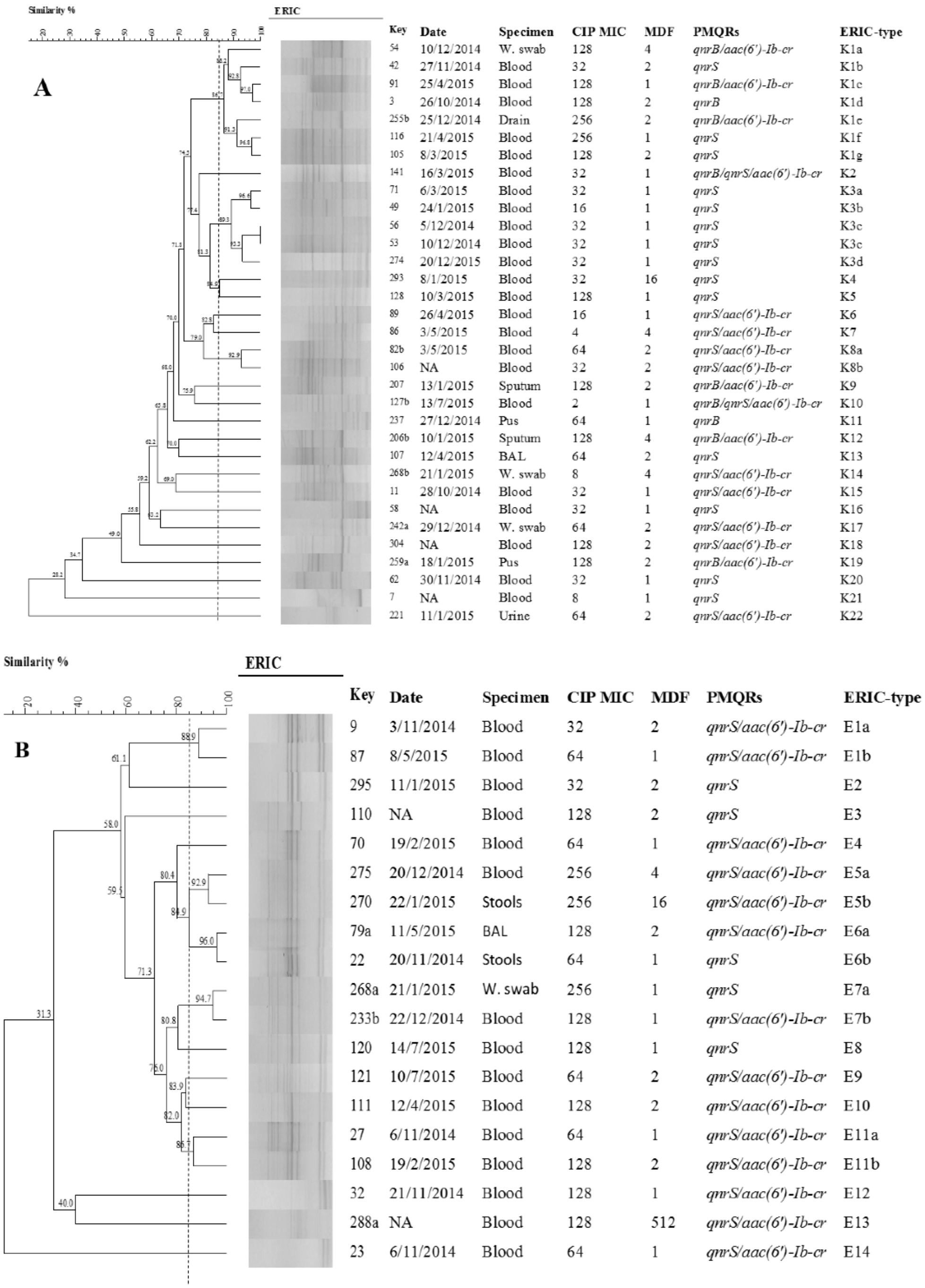


**Supplementary Figure S1. Dendrograms constructed using ERIC-PCR patterns of the *qnr*-positive isolates showing similarity percentages calculated by the dice similarity index. (A) *qnr*-positive *K. pneumoniae* isolates and (B) *qnr*-positive *E. coli* isolates.** BAL, bronchoalveolar lavage; CIP MIC, ciprofloxacin MIC (mg/L); PMQR, plasmid mediated quinolone resistance determinants; MDF, MIC decrease factor of ciprofloxacin. Dashed lines are hypothetical lines showing 85% similarity.
